# Lack or insufficient predialysis nephrology care worsens the outcomes in dialyzed patients – call for action

**DOI:** 10.1080/0886022X.2022.2081178

**Published:** 2022-06-01

**Authors:** Andrzej Milkowski, Tomasz Prystacki, Wojciech Marcinkowski, Teresa Dryl-Rydzynska, Jacek Zawierucha, Jacek S. Malyszko, Pawel Zebrowski, Konrad Zuzda, Jolanta Małyszko

**Affiliations:** a Fresenius Medical Care Polska S.A, Poznan, Poland; b Fresenius Nephrocare Polska sp. z o.o, Poznan, Poland; c 1st Department of Nephrology and Transplantology, Medical University of Bialystok, Białystok, Poland; d Department of Nephrology, Dialysis and Internal Medicine, Medical University of Warsaw, Warszawa, Poland

**Keywords:** Late referral, vascular access, hemodialysis, outcome, predialysis care

## Abstract

The phenomenon of patients with advanced renal failure accepted for dialysis at a late stage in the disease process (late referral [LR]) is known almost from the beginning of dialysis therapy. It may also be associated with worse outcomes. The aim of the study was to assess the effect of referral time on the outcomes, such as number of hospitalizations, length of stay, kidney transplantation, and mortality. A study of 1303 patients with end-stage renal failure admitted for dialysis in the same period in Fresenius Nephrocare Poland dialysis centers was initiated. The type of vascular access during the first dialysis was accepted as the criterion differentiating LR (*n* = 457 with acute catheter) from early referral (ER; *n* = 846). The primary endpoint was the occurrence of death during the 13-month observation. By the end of observation, 341 (26.2%) of patients died. The frequency of death was 18.1 for ER and 37.9 for LR per 1000 patient-months. It can be estimated that 52.1% (95% CI: 40.5–61.5%) of the 341 deaths were caused by belonging to the LR group. Patients from LR group had longer hospitalizations, more malignancies, lower rate of vascular access in the form of a–v fistula, higher comorbidity index. It seems that establishing a nephrological registry would help to improve the organization of care for patients with kidney disease, particularly in the pandemic era.

## 1. Introduction

The phenomenon of patients with advanced renal failure accepted for dialysis at a late stage in the disease process (late referral [LR]) is known almost from the beginning of dialysis therapy. Despite renal replacement therapy is widely available in many countries, LR continues to occur. Some problems associated with LR and in particular higher mortality of patients referred late, had been already described more than 30 years ago. A single center study showed that 41.8% of 55 patients were referred for repeated dialysis treatment too late [1]. Patients were found to suffer serious complications, such as pulmonary edema, severe hypertension, pericarditis, and greater mortality [2]. The scope of LR phenomenon in the literature varies widely. LR frequency is estimated at about 30%, although the distribution of the percentage of LR patients starting dialysis treatment varies from 24.1% [3] up to 80% [4]. The scope of LR in Poland has not been so far evaluated since only local or regional analyses were available. A total of 72 Fresenius Nephrocare dialysis centers in nearly all voivodeships (i.e., except for the Opolskie Voivodeship) provided an opportunity to conduct broader assessment of LR scope and impact. The aim of the study was to evaluate the effect of referral time on the outcomes, such as number of hospitalizations, length of stay, survival, kidney transplantation, and mortality.

## 2. Materials and methods

Based on the EuCliD electronic database, a list of patients starting dialysis treatment at the Fresenius Nephrocare dialysis centers in Poland in the period of one year was compiled. As EuCliD covers only the course of dialysis and does not contain obligatory information on pre-dialysis period, type of vascular access during the first dialysis was accepted as the criterion differentiating LR from early referral (ER), as by Pisoni et al. [5]. Patients starting dialysis with the use of both types (temporary and permanent) catheters were admitted as the LR patients. In pre-dialysis period, no attempt was made to establish a dialysis fistula in these patients. Patients who began dialysis over a native AVF or from graft or using a permanent catheter, when the attempt to establish a fistula was unsuccessful or there were contraindications (such as damaged vessels, cardiovascular complications, and contraindications for surgery) to the fistula creation were admitted as the ER patients. In some cases, when the fistula was not functioning or could not be punctured and permanent catheter placement was impossible for various mainly organizational reasons (availability of proper catheter, medical staff in place, and issues during implantation), the LR patients started dialysis using an acute catheter. In doubtful cases, the patients’ qualification for the above-mentioned groups was verified directly in the dialysis center.

Data was collected for each patient prior to the first visit: vascular access (temporary CVC permanent catheter, AV fistula, and graft), age, BMI, systolic and diastolic arterial pressure, laboratory tests: eGFR, urea concentration, 50 Hb, phosphate, albumin, PTH, glucose, accompanying diseases: diabetes, hypertension, malignancy, Charlson comorbidity index, and dose of ESA determined in the first month of dialysis (darbopoetin doses converted to erythropoietin alfa). During 13-month observation the following data was collected: number of hospitalizations and length of stay, patients’ outcomes, i.e., survival, kidney transplantation, and mortality. The collected data was subjected to statistical analysis. Since only the available data from the EUCLiD system was analyzed, written consent was not needed in accordance with the regulations in force at Fresenius Nephrocare in Poland and Ethics Committee at the Warsaw Medical University.

The results are presented as percentage for categorical values, mean value with one standard deviation in case of variables normally distributed. For non-normally distributed variables median and minimum–maximum were presented. Data given was analyzed using Statistica version 13.1 (Tulsa, Oklahoma, USA). For statistical significance assessment T-Test, Chi-Square, and Mann–Whitney tests were used accordingly, Kruskal–Wallis ANOVA for repeated measurements were used in statistical analysis with *p* < 0.05 considered statistically significant.

Survival analysis was performed and presented Kaplan–Meier’s survival curves and Nelson–Aalen’s cumulative risk chart. In addition, 61 Cox’s proportional hazard analysis was performed as well as Schoenfeld residues analysis to assess the impact of belonging to one 62 of the groups (LR or ER) on the risk of death.

## 3. Results

During one period, dialyses of 1303 patients with end-stage renal failure were initiated in 65 Fresenius Nephrocare Poland dialysis centers. According to the above criteria, 846 patients were ‘Early Referral’ and 457 patients were group ‘Late Referral’. In some cases, with created a–v fistula but not either functioning or unaccessible to puncture, acute catheter was placed (sometimes in particular during evening or weekends permanent catheters are not inserted due to logistics). Therefore, 19.7% of patients in ER group started HD with acute catheter. Every case was verified in each center and centrally in the Euclid system (Table 1).

**Table 1.** The table contains a comprehensive data comparison in both groups of patients.

Feature	Early referral [*N* = 846]	Late referral [*N* = 457]	*p*
Sex [men/women]	508/338 (60.0/40.0%)	225/201 (55.9/44.1%)	0.15
Age [years] Age > 65 years [*N* (%)]	65 ± 15	67 ± 14	0.06
	451 (53.3%)	255 (55.8%)	0.39
BMI [kg/m^2^]	27.9 ± 6.3	27.1 ± 6.7	<0.05
BMI <18.5 kg/m^2^ [*N* (%)]	23 (3.0)	18 (4.8)	<0.05
BMI 18.5 − 24.9 kg/m^2^ [*N* (%)]	246 (31.8)	151 (39.9)	<0.05
BMI 25 − 29.9 kg/m^2^ [*N* (%)]	256 (33.1)	104 (27.5)	<0.05
BMI ≥ 30 kg/m^2^ [*N* (%)]	248 (32.1)	105 (27.8)	<0.05
SBP [mmHg]	134.3 ± 21.5	132.7 ± 23.8	0.25
DBP [mmHg]	75.1 ± 12.5	75.0 ± 12.4	0.87
Vascular access	–	–	–
Acute catheter [*N* (%)]	164 (19.7%)	269 (59.6%)	<0.001
Tunneled catheter [*N* (%)]	248 (29.7%)	180 (39.9%)	<0.001
Arteriovenous fistula [*N* (%)]	419 (50.2%)	3 (0.7%)	<0.001
Arteriovenous graft [*N* (%)]	6 (0.7%)	0	–
Laboratory data	–	–	–
Hb [g/dL]	9.65 ± 1.44	9.39 ± 1.49	<0.01
Hb ≥ 10 g/dL [*N* (%)]	276 (38.2%)	97 (26.4%)	<0.001
P [mg/dL]	4.87 (3.90-6.10)	5.13 (4.03–6.18)	0.26
P < 5 mg/dL [*N* (%)]	366 (53.2%)	151 (44.9%)	<0.05
PTH [pg/mL]	337.7 (188.7-539.4)	290.7 (161.3–493.2)	0.08
PTH <130 pg/mL [*N* (%)]	100 (15.8%)	55 (18.2%)	0.36
PTH > 600 pg/mL [*N* (%)]	132 (20.9%)	52 (17.2%)	0.19
Albumins [g/L]	35.9 ± 5.9	32.7 ± 6.0	<0.001
Albumins ≥ 40 g/dL [*N* (%)]	177 (28.3%)	32 (10.5%)	<0.001

BMI: body mass index; Hb: hemoglobin; P: phosphate; PTH: parathyroid hormone; SBP: systolic blood pressure; DBP: diastolic blood pressure

### 3.1. Assessment of the impact of observation time on survival

**Figure 1.**
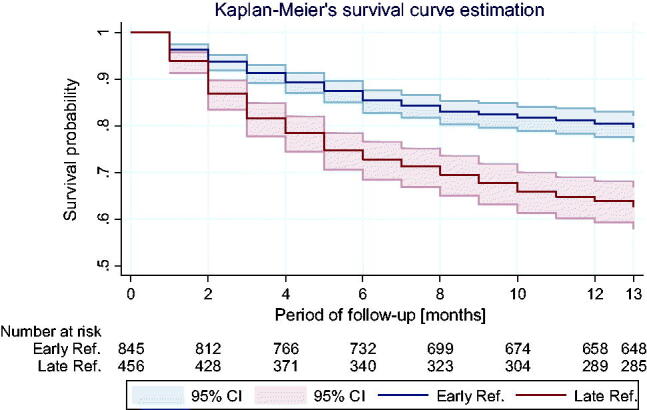
Kaplan–Meier’s survival curves in both ER and LR groups.

Below figure shows Kaplan–Meier’s survival curves in both ER and LR groups (Figure 1).

**Figure 2.**
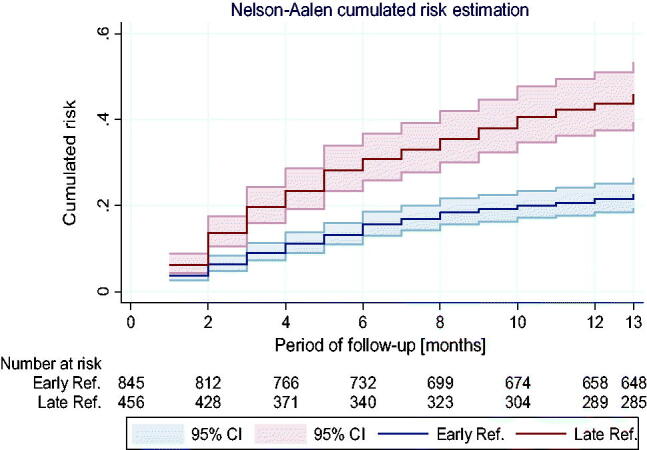
Nelson–Aalen’s cumulative mortality hazard chart presents curves of the cumulative hazard function in the ER and LR group.

**Figure 3.**
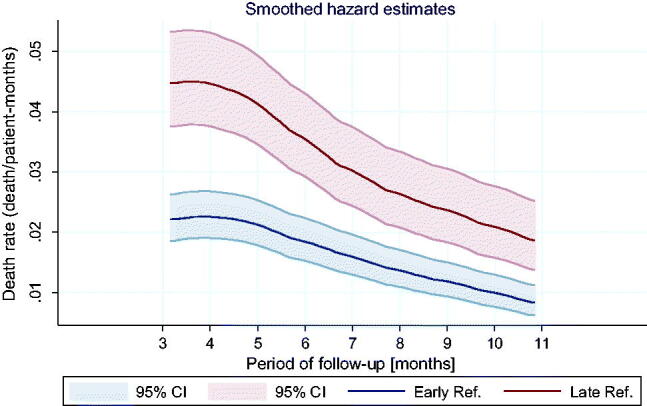
Smoothed curves of hazard function (death rate depending on the observation time) in ER and LR groups.

The survival curves diverge statistically starting from the 6th month of observation. In their course, there is a statistically significant difference (log-rank test: *χ*^2^ = 45.5, *p* < 0.001) (Figures 2 and 3).

Cox’s proportional hazard analysis showed that patients with LR had significantly higher risk of death than the patients in the ER group (HR = 2.02, 95% CI: 1.64–2.50, *p* < 0.001). Based on the Schoenfeld residues analysis, proportionality of assumptions fulfillment has been demonstrated (*p* = 0.98), which means that: the impact of belonging to one of the groups (LR or ER) on the risk of death is independent of time of observation and the frequency of death incidences decreases during observations in both groups, however, in the LR group, the risk remains significantly higher throughout the observation period.

### 3.2. Assessment of the impact of age on the survival

LR patients in the group below 65 years had more than twice the risk of death in comparison to the ER group (relative death rate = 2.44; 95% CI: 1.65 − 3.62; *p* < 0.001). LR patients in the group above 65 years had almost twice the risk of death in comparison to the ER group (relative death rate = 1.94; 95% CI: 1.48 − 2.53; *p* < 0.001).

Age-corrected (Mantel–Haenschel’s method) relative death rate was 2.09 (95% CI: 1.69 − 2.58) and the homogeneity test did not show that the age below and = >65 years was a modifying effect and influenced the relative frequency of deaths (*χ*^2^ = 0.97, *p* = 0.326).

**Figure 4.**
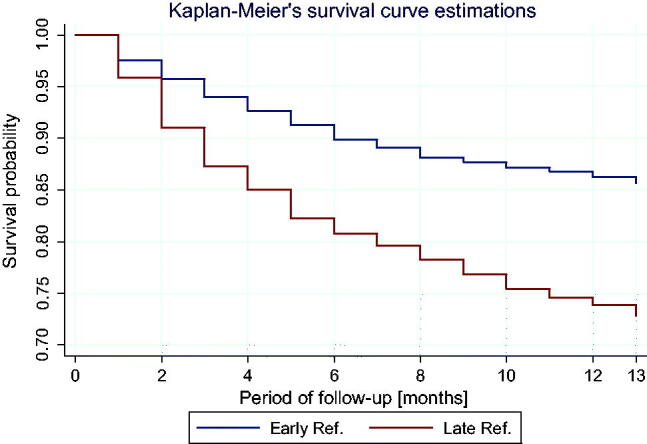
Kaplan Meier’s survival curves in both ER and LR groups, corrected in relation to the patients’ age.

Below figure shows Kaplan–Meier’s survival curves in both groups of patients, corrected in relation to the patients’ age (Figure 4).

**Figure 5.**
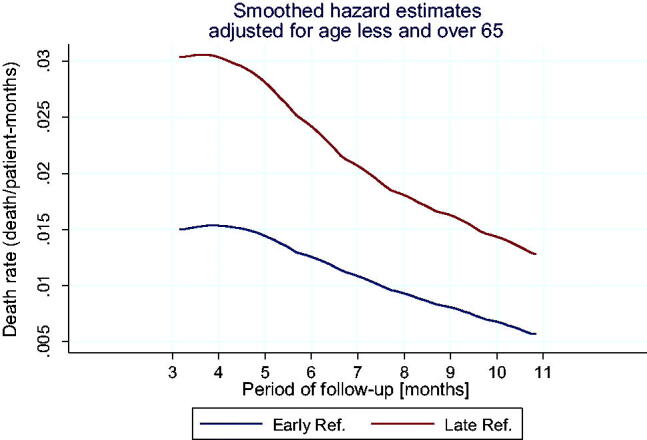
Age-corrected curves of hazard function (death rate depending on the observation time) in ER and LR groups frequency of death incidences decreases during observations in both groups, however, in the LR group, the risk remains significantly higher throughout the observation scope. Cox’s proportional hazard analysis based on the age-correction showed that the patients in the LR group had significantly higher risk of death than ER patients (HR = 2.01; 95% CI: 1.63–2.49; *p* < 0.001).

A statistically significant difference in the course of survival curves between the groups was found (log-rank test: *χ*^2^ = 45.1 *p* < 0.001) and the survival curves diverge statistically starting from the 6th month of observation (Figure 5).

Based on the analysis of Schoenfeld’s residuals, the proportionality of hazard assumptions has been shown (*p* = 0.933), which means that the group’s impact on the risk of death is independent of the observation time.

### 3.3. Morbidity index

**Figure 6.**
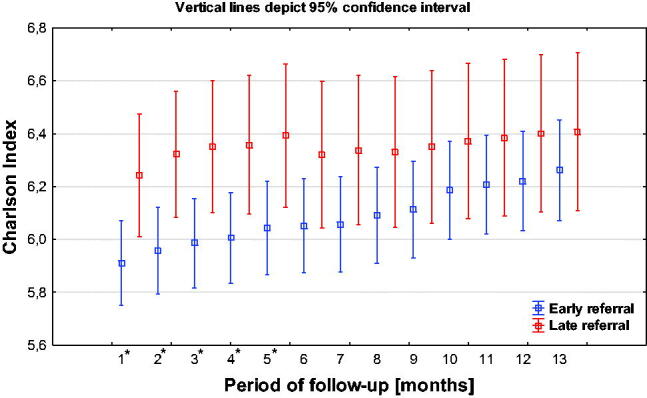
Charlson index in the compared ER and LR groups.

The chart below presents the Charlson Index in both compared groups (Figure 6).

Statistically significant differences in morbidity remain up to 5th month. In subsequent months of observation, the differences in the Charlson index are gradually decreasing (Table 2).

**Table 2.** The Comparison of the initial eGFR values and the occurrence of diabetes, hypertension, and neoplasms in ER and LR groups.

Feature	Early referral [*N* = 846]	Late referral [*N* = 457]	*p*	OR LR *vs.* ER ± 95% CI
eGFR [mL/min/1.73 m^2^]	10.1 ± 4.2	9.2 ± 4.3	**<0.001**	–
eGFR <6 [mL/min/1.73 m^2^]	108 (13.0%)	102 (22.5%)	**<0.001**	1.94 (1.44 − 2.61)
Diabetes [*N* (%)]	353 (47.8%)	178 (38.9%)	0.32	–
Hypertension [*N* (%)]	730 (86.3%)	358 (78.3%)	**<0.001**	0.57 (0.43 − 0.77)
Neoplasms [*N* (%)]	129 (15.3%)	92 (20.1%)	**<0.05**	1.40 (1.04 − 1.88)

Average values + standard deviation. Bold values are statistically significant.

At the beginning of the observation in the LR group, the estimated glomerular filtration rate (eGFR) was significantly lower and the malignancy was more frequent. In the ER group, however, hypertension was significantly more frequently diagnosed and diabetes, at the same time more often (Table 3).

**Table 3.** Single- and multi-component analysis of mortality in the entire studied population with consideration of time of including in hemodialysis.

Feature	HR	−95% CI	+95% CI	*p*
Single-component analysis	–	–	–	–
ER *vs.* LR	0.49	0.40	0.61	**<0.001**
Age [years]	1.035	1.026	1.044	**<0.001**
Diabetes	1.03	0.83	1.28	0.75
Hypertension	0.47	0.37	0.60	**<0.001**
Neoplasms	2.34	1.86	2.96	**<0.001**
eGFR <6 [mL/min/1.73 m^2^]	1.30	0.99	1.71	0.054
Multi-component analysis	–	–	–	–
ER *vs*. LR	0.54	0.44	0.67	**<0.001**
Age [years]	1.034	1.025	1.043	**<0.001**
Diabetes	1.11	0.88	1.39	0.37
Hypertension	0.58	0.45	0.75	**<0.001**
Malignancy	1.87	1.46	2.39	**<0.001**
eGFR <6 [mL/min/1.73 m^2^]	1.19	0.90	1.57	0.23

Bold values are statistically significant.

The effect on the mortality in the entire studied population depends upon time of referral, age, comorbidities, such as hypertension and malignancy.

On the other hand, neither the presence of diabetes nor profound impairment of renal excretion (eGFR < 6 mL/min) affect mortality during the 13-months observation.

### 3.4. Hospitalizations

**Figure 7.**
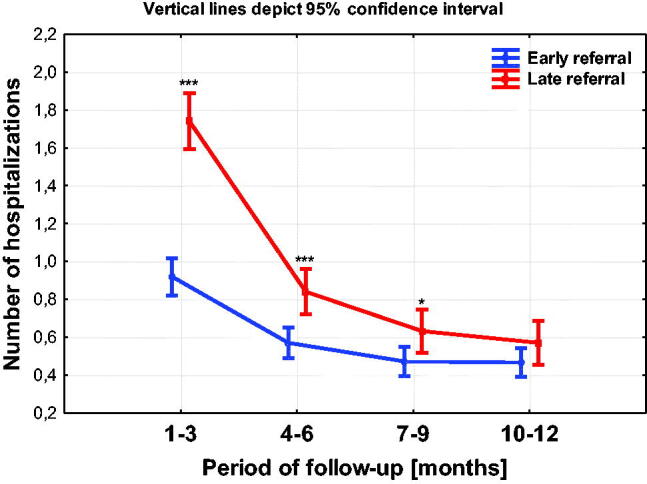
Number of hospitalizations in the compared groups. *p < 0.05; **p < 0.01; ***p < 0.001.

The chart below shows the number of hospitalizations in both groups during the 13-month observation (Figure 7).

Number of hospitalizations in both groups decreases with time. By the 9th month, the number of hospitalizations in the LR group is statistically significantly higher than in the case of patients from the ER group. In the following months, the difference between the LR and ER groups is maintained, although it is no longer statistically significant (Table 4).

**Table 4.** Hospitalization duration (in days).

Observation time	Early referral [*N* = 846]	Late referral [*N* = 457]	*p*
1–3 months	12.6 ± 13.6	15.5 ± 14.1	<0.01
4–6 months	9.3 ± 11.0	11.8 ± 14.6	0.06
7–9 months	9.1 ± 11.0	11.4 ± 15.6	0.16
10–12 months	10.7 ± 13.0	10.8 ± 14.2	0.97

Average value and standard deviation.

In the early period of the observation (from 1st to 3rd month) the length of hospitalization in the LR group was statistically significantly higher (*p* < 0.01) compared to the ER group. In the following months, times of hospitalization in both groups gradually approached each other although they were still slightly longer in the LR group.

### 3.5. Vascular access

**Figure 8.**
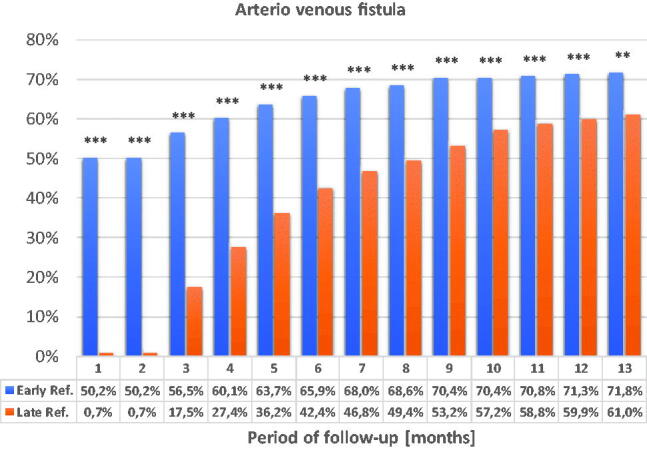
Percentage of patients with arteriovenous fistula during the 13-month observation. *p < 0.05; **p < 0.01; ***p < 0.001.

**Figure 9.**
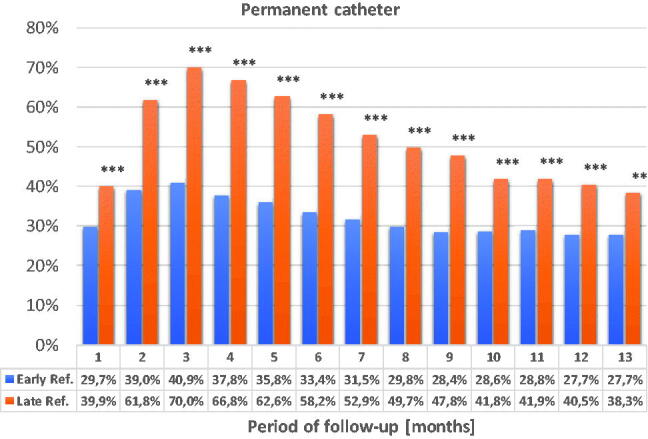
The use of a tunneled catheter as vascular access during the 13-month observation. *p < 0.05; **p < 0.01; ***p < 0.001.

The differences in the use of arteriovenous fistula as vascular access were statistically significant throughout the 13-month observation period (Figures 8 and 9).

The frequency of using the tunneled catheter decreases in both groups but catheters are used significantly more frequently in the LR group throughout the observation period.

### 3.6. Hemoglobin concentration and ESA dosing

**Figure 10.**
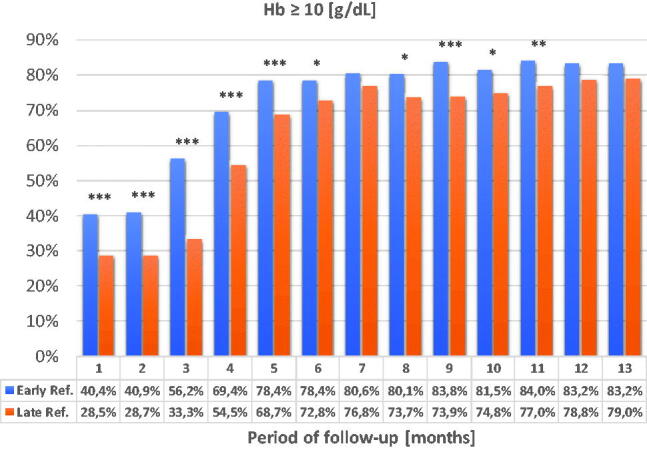
Differences in Hb concentrations between LR and ER groups in the subsequent months of observation. *p < 0.05; **p < 0.01; ***p < 0.001.

**Figure 11.**
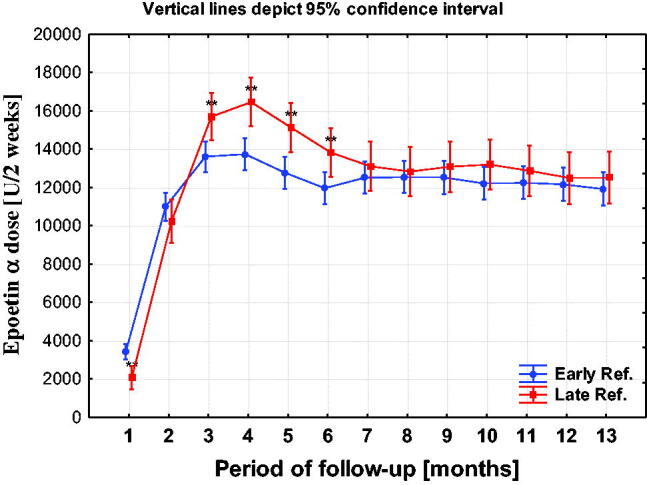
ESA dosing in ER and LR groups in the following months of observation. *p < 0.05; **p < 0.01; ***p < 0.001.

Differences in Hb concentrations in favor of the ER group remained until the end of the 13-month observation, although in the 7th month and in the last 2 months of observation, the differences lost their statistical significance (Figures 10 and 11).

The dosage of erythropoietin was significantly higher in the LR group up to the 6th month of observation and remained, with non-significant differences, until the end of the observation.

### 3.7. Phosphatemia and PTH

**Figure 12.**
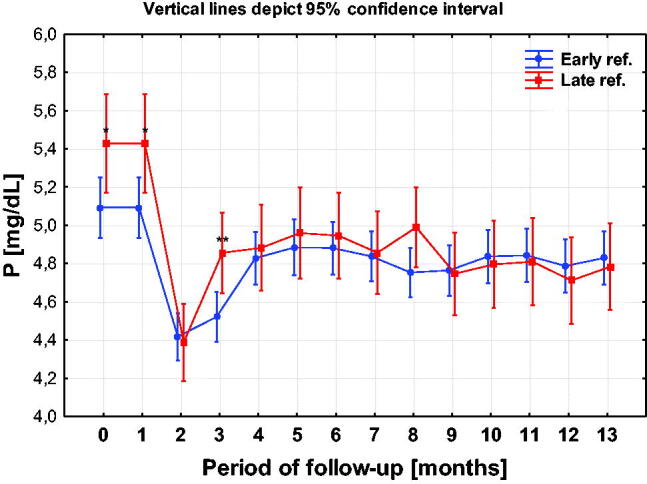
Concentrations in phosphates in the compared ER and LR groups.

Conclusion: statistically significant differences disappear in the 4th month of observation but remain, at least at the statistically insignificant level, up to the 8th month of observation. In the remaining 5 months, up to the end of the observation, the phosphatemia in the ER group is slightly higher than in the LR but remains within the normal range. It may be, at least partially, associated with the worse state of nourishment of the LR patients described below (Figure 12, Table 5).

**Table 5.** PTH concentration in ER and LR groups during the entire observation.

Observation time	Early referral [*N* = 846]		Late referral [*N* = 457]	
	PTH [pg/mL]	PTH 130–600 pg/mL [*N* (%)]	PTH [pg/mL]	PTH 130–600 pg/mL [*N* (%)]
1st month	337.7* (188.8 − 539.4)	400 (63.3)	290.8 (161.3 − 493.2)	194 (64.2)
2nd month	330.9* (176.2 − 567.6)	137 (65.2)	308.7 (128.8 − 506.5)	58 (54.7)
3th month	289.8* (161.6 − 549.7)	80 (57.1)	260.7 (125.8 − 397.9)	45 (60.0)
4th month	370.6*** (175.9 − 669.4)	83 (55.3)	236.6 (138.3 − 435.2)	40 (62.5)
5th month	332.8* (191.9 − 634.0)	83 (55.0)	297.3 (159.2 − 530.6)	35 (61.4)
6th month	382.1*** (199.7 − 634.7)	80 (54.4)	235.3 (141.7 − 393.5)	47 (64.4)
7th month	303.2** (147.2 − 468.3)	100 (61.7)	209.9 (100.5 − 366.7)	39 (51.3)
8th month	319.6*** (156.4 − 502.8)	111 (61.0)	208.8 (101.6 − 457.1)	32 (48.5)
9th month	283.6* (149.3 − 501.2)	94 (59.5)	265.0 (167.0 − 561.6)	32 (56.1)
10th month	308.3 (169.2 − 602.4)	94 (56.0)	331.5 (156.7 − 545.7)	44 (61.1)
11th month	335.9*** (193.5 − 571.0)	91 (63.2)	255.9 (154.6 − 483.1)	39 (61.9)
12th month	400.7*** (167.5 − 583.7)	94 (59.5)	215.7 (148.8 − 390.6)	42 (65.6)
13th month	323.7*** (187.0 − 542.9)	119 (63.0)	230.0 (123.0 − 389.1)	45 (62.5)

ER *vs.* LR. (U Manna–Whitney Test) = *p < 0.05; **p < 0.01; ***p < 0.001.

The average concentration of PTH was higher in the ER group (bolded) during almost the entire observation period (except for the 10th month) but not exceeding 600 pg/mL. This may indicate that the LR group may have been at greater risk of developing adynamic bone.

### 3.8. Nutritional condition

Nutritional condition was assessed by marking BMI and albumins concentration.

#### 3.8.1. Albumins

Up to the 3rd month of observation, LR patients were characterized by hypoalbuminemia. Albumin concentration was higher in the ER group during the entire observation period (except for the 11th month) and the difference was statistically significant until the 5th month of observation (Table 6).

**Table 6.** Albumin concentration in ER and LR groups in particular months of observation.

Feature	Early referral [*N* = 863]	Late referral [*N* = 472]	*p*
1st month	35.83 + 5.94	32.87 + 5.95	**<0.001**
2nd month	35.55 + 5.99	33.44 + 5.75	**<0.001**
3th month	36.81 + 5.64	34.32 + 5.72	**<0.001**
4th month	37.90 + 4.56	35.64 + 5.82	**<0.001**
5th month	37.95 + 5.54	36.42 + 6.32	**<0.05**
6th month	38.02 + 4.94	37.48 + 5.19	0.35
7th month	38.49 + 4.62	37.38 + 5.75	0.07
8th month	38.89 + 4.60	37.41 + 6.08	0.02
9th month	38.46 + 4.70	37.99 + 4.70	0.39
10th month	38.87 + 4.09	38.41 + 4.88	0.38
11th month	38.90 + 4.40	39.19 + 4.82	0.61
12th month	38.67 + 4.60	38.35 + 5.81	0.57
13th month	39.01 + 4.21	38.48 + 4.28	0.29

Bold values are statistically significant.

#### 3.8.2. BMI

**Figure 13.**
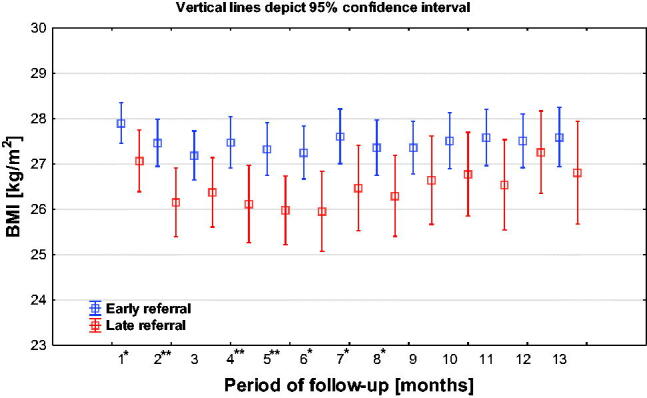
BMI index during the 13-month observation.

In the first months of observation, LR patients were characterized by a significantly worse condition of nutrition (BMI index was significantly lower in the LR group up to the 8th month of observation). The difference in favor of the ER group was visible until the end of the observation, although in the last months it was no longer significant (Figure 13).

## 4. Discussion

Referring the patients with terminal renal failure to a nephrologist too late is a well-known phenomenon. The definition of LR is not unified. Time frames for LRs differ significantly in numerous publications, most often this group includes patients referred to the nephrologist less than 3 months prior to starting of the dialysis [6–9] – although a much longer time frame is given, e.g., up to 6 months [10] and even up to 1 year [11]. On the other hand, the introduction of term *ultralate* is also suggested – up to 3 months [11] or even up to 1 month [9]. In general, LR is considered when patient is seen by nephrologist within one to six months of the requirement for kidney replacement therapy [12]. In various studies in the United States, 25–50% of patients started chronic dialysis within one month of their first nephrology visit [13–15]. Similarly, 25% of patients who required dialysis within one month of the first visit to a nephrologist started renal replacement therapy in Paris, France [16], and 58% in Sao Paulo, Brazil [17]. Ifudu et al. [18] reported that some patients received no medical care at all. The frequency of LR reported in the literature is very diverse: 80% [4], 60.3% [6], 57.3% [10], 41% [8], 37.6% [11], 34% [7], 33% [19], and 24.1% [3] and in general studied groups are relatively small.

In the presented analysis, it was determined that LR group constituted 35.7% of the total of 1303 patients dialyzed at Fresenius dialysis centers. As mentioned above, in the absence of information on a possible nephrological care in the EuCliD database in the pre- dialysis period, the criterion for belonging to the LR or ER group was the type of vascular access during the first hemodialysis. Similar criteria were adopted, among others by Pisoni et al. [5].

The frequency of LR phenomenon in Poland, on a national scale, is not known. Only partial data is available: 49% of LR patients from the region of south-eastern Poland prior to reporting to the dialysis center had symptoms of uremia, 20% had to start dialysis within 1 month [20]. The same percentage − 49% was found among 626 patients starting dialysis in 2012 [21] but this data is limited, as it comes from the 25 Diaverum Dialysis Centers in Poland, Hungary, and Romania.

It should be noted that LR observations generally do not include a large group of patients with deteriorated kidney function, in which nephrological intervention leads to improvement of renal excretion, and treatment of comorbidities. Therefore, the above-mentioned criteria for the classification of patients lead to underestimation of the real impact of LR on the prognosis. It is known that cardiovascular disease in patient in Stage 3 CKD is highly likely to lead to the patient’s death before he/she reaches the level of renal failure requiring dialysis [16].

The presence of the LR phenomenon has been of great interest for years because LR group of patients is burdened with a particularly high risk of death [3,8–10,19,22–27]. It was demonstrated that during the first year of dialysis, patients’ mortality in LR group is much higher than in patients belonging to the ER group (28.9 *vs.* 8.5%) [28] and may remain at a level higher than in ER group up to 5 years after starting the dialysis [29].

The survival analysis presented in this study is consistent with the above observations: the risk of death of LR patients was about twice as high as in the case of patients in ER group. The frequency of deaths decreased during observations in both groups, however, in LR group, the risk remained significantly higher throughout the observation period. The age of the patients was not a factor modifying the risk of death. The influence of belonging to the ER or LR group on the risk of death was also independent of the time of observation.

The high mortality of LR patients may be caused by numerous metabolic, hormonal, and hematological disorders, such as anemia, malnutrition, hyperparathyroidism, hyperphosphatemia, hypocalcemia, hypoalbuminemia, hypertension, congestive heart failure, and infections. Each of the above-mentioned factors can be modified in the pre-dialysis period by appropriate proceedings [30].

The morbidity in the examined patient groups was analyzed using both the Charlson index and the assessment of presence of diseases that have a decisive influence on the patients’ fate: hypertension, diabetes, and neoplasm.

The Charlson index was statistically significantly higher in the LR group up to the 5th month of observation. In the following months it was still higher in the LR group, although the differences were statistically insignificant. The probability of survival was dependent on the Charlson Index class.

LR patients – in comparison with ER – started dialysis with significantly lower eGFR and what is surprising – they were less likely to have diabetes (statistically insignificant) and hypertension (non-statistically significant). Perhaps this could be a sign of the effectiveness of the automatic eGFR calculation at each determination of creatinine concentration as well as more intensive medical care of patients with diabetes.

In the group of the LR patients, statistically more frequent occurrence of neoplastic diseases was found.

Both single-component and multi-component analysis showed that increased mortality was in favor by LR group membership, age, hypertension, and cancer. On the other hand, neither the presence of diabetes nor profound impairment of renal excretion (eGFR < 6 mL/min) affected mortality during the 13-month observation.

LR means more frequent and longer hospitalizations for patients, also associated with the presence of comorbid conditions [4,9]. The analysis of hospitalization in this observation showed that the number of hospitalizations in both groups decreased with the passage of time and up to the 9th month the differences were statistically significant to the detriment of LR group. In the following months, the difference between the LR and ER groups is maintained, although it is statistically insignificant. However, the duration of hospitalization only in the initial period of observation (from 1 to 3 months) in the LR group was statistically significantly longer (*p* < 0.01) compared to the ER group. In the following months, duration of hospitalization in both groups gradually approached each other although they were still slightly longer in the LR group.

Dialysis using arteriovenous fistula dominated throughout the observation period in the ER group. Over time, the frequency of using the arteriovenous fistula also increased in the LR group but until the end of the observation the statistically significant difference remained. In the LR group, arteriovenous fistulas were performed in three patients in the first month of observation. Other LR patients had been performed the arteriovenous fistulas only in the third month.

Hb concentrations were higher in the ER group and this difference was maintained throughout the observation period. The differences were statistically significant with the exception of the last two months. The dosage of erythropoietin was significantly higher in the LR group up to the 6th month of observation and remained with non-significant differences until the end of the observation.

The phosphate concentrations in LR patients were statistically significantly higher up to the 3rd month of observation. The difference – although insignificant – remained until the 6th month. Differences disappeared during further observation. PTH concentrations were significantly higher in the ER group.

The nutritional condition of patients in the LR group – assessed by BMI and albumin concentration – was significantly worse than the ER group.

Dialysis access in LR patients was provided by vascular catheters constituting a significant source of infectious complications. It should be noted that this group of patients is devoid of a number of significant possibilities: decision on the optimal method of dialysis – dialysis of LR patients starts with the use of catheter hemodialysis and the patient almost never starts PD dialysis [31], evaluation for kidney transplantation after starting the dialysis is important (as much as fivefold) to reduce the chance of kidney transplantation [28], undergoing pre-dialysis education which has a significant impact on the patient’s future, including the acceptance of the renal replacement therapy method [32–38].

The LR phenomenon means generally higher system costs for the society, including costs related to hospitalizations or treatment of anemia [4,11,28,39].

In turn, ER means for the society: a delay in starting the dialysis which means the postponement of costs related to dialysis in time and reduction of costs associated with hospitalizations which are less frequent than in the patients with LR. The causes of LR are complex – one can cite, above all, the lack of social awareness of dangers of kidney diseases, and their excretory function [40].

In the NEFROTEST program (campaign organized by NEFRON – the nephrological section of the Polish Chamber of Commerce Medical Association for the dialysis centers owners in Poland; besides the informative and educational part, it also included free blood creatinine determination) – out of 22,113 voluntarily tested, none of the 2343 persons, who were diagnosed with chronic kidney disease (CKD), knew about their kidney disease (unpublished data).

In the USA, delayed referral to the nephrologist usually takes place when the GP is an internist, whereas the delay is less frequent when it is a family doctor or GP [41].

European observations are similar – LR occurs most often when the internist or other specialist (e.g., diabetology or cardiology) is the referring physician, not the first-contact physician [42].

Situations are also described when a patient with terminal renal failure had the first contact with the nephrologist only in the dialysis center. When analyzing data of 443,761 patients who started dialysis in the US in the years 2006–2010, Gillespie et al. report that as many as 33% of patients referred for the dialysis were not undergoing nephrological care [19].

In the DOPPS study, about 20% of patients starting HD in countries participating in this program did not see a nephrologist prior to the dialysis and monitoring of the incidence and treatment of diabetes, hypertension, dyslipidemia, anemia, malnutrition, or bone disorders was far from the recommendations [38].

It was also proved that LR patients who are not under nephrological supervision are much less likely to receive RAAS blocking drugs (LR − 32% and ER − 57%) or alphacalcidol (LR − 5% and ER − 30%) [31,42].

It turns out, however, that even the visit of a patient to the nephrologist does not always mean proper care over the patient. In the complementary project, the START study among 436 patients starting the dialysis, 56.4% of patients monitored over 12 months by nephrologists had a ‘suboptimal start of dialysis’ (i.e., start of dialysis in hospital conditions and/or a catheter as a vascular access), including, among others: 31.25% – delay due to the patient’s fault, 31.25% – exacerbation of CKD

, 16.41% – delay caused by the surgeon, 8.59% – late decision to start HD, and − 12.5% = other causes [43].

When considering the problem of late visits of patients to the nephrologist, the problem of accessibility to specialist nephrological care should be taken into account. This may be a problem of insufficient number of nephrologists in relation to the needs as well as the location of nephrological clinics which should be correlated with the size of the population served.

Early detection of patients eligible for referral to a nephrologist requires diversified activities. It calls for: systematic management and repeating media actions informing the society about the importance of early diagnosis of CKD, carrying out public campaigns that give the possibility to detect CKD in spontaneously visiting persons. Examples of such activities are as follows: the KEEP program widely runs in the USA – points are organized in public locations. One person’s questionnaire, blood and urine tests, and medical consultations take up to 45 min [44]. NEFROTEST program of the Nephrology Section of the ‘Nefron’ Polish Chamber of Commerce Medical. Among the 22,113 people who so far voluntarily and spontaneously reported for the tests, the presence of CKD was found in 2343 people or 10.04% (unpublished data). It should be emphasized that none of these people knew about their kidney disease. Incidental test campaigns carried out in limited numbers [45], automatic calculation of eGFR in every case of creatinine determination (introduced in Poland about 10 years ago). It was described that after implementation of this rule, the frequency of first visits at the nephrologists increased by 68.4% [46] but the legitimacy of referrals worsened. An example is referring the elderly patients with a moderate reduction of GFR to approximately 50–60 mL/min/1.73 m^2^ without any other symptoms of kidney disease [46–49], continuous cooperation with diabetology and cardiology clinics whose patients have a particularly high risk of coexistence of CKDs, distribution of nephrological clinics should be correlated with the size of the population served. Currently, there are 108,000–264,000 inhabitants per nephrology clinic in Poland. In the last quarter of 2019, according to NHF’s data, 290 nephrological clinics had contracted nephrological advice. In almost 50% of outpatient clinics the waiting time for the first appointment exceeds 3 months and in 5% of clinics exceeds 1 year. Low and inadequate valuation of procedures in nephrological clinics. The value of the NHF contract for nephrological advice per 1 inhabitant per year varies from 0.33 to 0.93 PLN (i.e., 0.08–23 euro). It seems that the establishment of a nephrological registry would help to improve the organization of care for patients with kidney disease. Introduction of a ‘pay-for-performance’ program in Taiwan for the care of patients with advanced renal disease is worth noting. It turns out that both the risk of starting the dialysis and the risk of death are equally measurable, long-term economic benefits have been achieved [50].

We are aware of the limitations of our study. As stated before, no data on a possible nephrological care in the EuCliD database in the predialysis period were available for many patients. It may be due to the fact that patients were followed by primary care physicians, waiting time for nephrology consults was too long as well as access to nephrology services was not optimal. In addition, due to the lack of the central system unable us to get the information of the previous nephrology care. Some of the patients were lost to follow up in the nephrology care and came too late to have conservative therapy but start dialysis instead or the were admitted to the hospital due to their clinical conditions and begun renal replacement therapy and then were transferred to outpatient dialysis unit for chronic HD. However, in this study, we have followed relatively large and homogeneous population in regard to care.

## 5. Conclusions

LR is a huge burden for dialysis units. There is a term ‘crushlanders’ used to describe LR patients. In 2005, Wauters et al. [51] discussed why patients with progressing kidney disease are referred late to a nephrologist. On the basis of our results, this discussion is still valid and problem unsolved. The situation has worsened during the COVID-19 pandemic. We are also aware that many of the achievements in nephrology care were lost in the pandemic as many patients were deprived of the permanent care, some of them feared to come for checkups, and telemedicine was not always helpful. LR to a nephrologist in progressing CKD is clearly detrimental to patients, the medical community, and the healthcare system. In addition, convincing health care administrators to introduce simple measures for early detection and treatment of CKD is prerequisite. This approach is by far more cost-effective than any expensive ESRD treatment. Establishing of a nephrological registry and improved interdisciplinary cooperation with primary care physicians in particular, would help to improve the organization of care for patients with kidney disease [52]. Setting-up a consultation network at a regional or local level appears as one of the most urgent and effective steps.

### Institutional review board statement

Ethical review and approval were waived for this study, due to collection of the retrospective data.

## Abbreviations

ANOVA: analysis of variance
BMI: body mass index
CI: confidence interval
CKD: chronic kidney disease
CVC: central venous catheter
DBP: diastolic blood pressure
eGFR: estimated glomerular filtration rate
ER: early referral
ESA: erythtropoietin stimulating agent
ESRD: end-stage renal disease
EuCliD: European Clinical Dialysis electronic database
GP: general practitioner
Hb: hemoglobin
HD: hemodialysis
LR: late referral
NHF: National Health Found
P: phosphate
P LN: polish currency zloty
P T H: parathyroid hormone
RAAS: renin angiotensin aldosterone system
SBP: systolic blood pressure
